# Pain in Chronic Medical Illness

**DOI:** 10.1155/2013/615967

**Published:** 2013-01-20

**Authors:** Justin Brown, Jarred Younger, Alok Madan, Jeffrey Borckardt

**Affiliations:** ^1^Department of Biology and Environmental Science, Simpson College, 701 North C Street, Indianola, IA 50125, USA; ^2^Department of Anesthesia, School of Medicine, Stanford University, Suite 207F, 780 Welch Road, Palo Alto, CA 94040, USA; ^3^Department of Psychiatry and Behavioral Science, College of Medicine, Medical University of South Carolina, 67 President Street, IOP 5-North, 507, Charleston, SC 29425, USA

It has recently been reported that chronic pain affects 43% of American, approximately 100 million adults in 2012 (Tsang and colleagues, The Journal of Pain 9(10): 883–891, 2008); accordingly, it remains essential that research continue to advance the current ability to assess and manage pain. Here, in this special issue, we highlight the impact of pain in chronic medical illness and present a body of research that addresses areas where data are currently lacking. Specifically, we present articles that characterize pain in cardiovascular disease, pulmonary disease, breast cancer, gastric bypass surgery, neurofibromatosis, low back pain, and postherpetic neuralgia. Importantly, these studies characterize pain in underrepresented populations including adolescents and surgical patients, and in diverse populations that include individuals from the United States, Iran, and France. Several commonalities arise involving an association between obesity and pain, a call for increased patient education, and a call for continuing education in pain for health care professionals.

Included, you will find the following ten articles: (1) *“Prevalence of chest pain, depression, somatization, anxiety, global distress, and substance use among cardiac and pulmonary rehabilitation patients”* by E. Serber and colleagues, (2) “*Pain narratives in breast cancer survivors”* by P. Peretti-Watel and colleagues, (3) “*Presurgical weight is associated with pain, functional impairment, and anxiety among gastric bypass surgery patients*” by S. Wedin and colleagues, (4) “*Physical, cognitive, and psychosocial predictors of functional disability and health-related quality of life in adolescents with neurofibromatosis-1*” by M. Garwood and colleagues, (5) “*The pain crisis: what it is and what can be done*” by B. Sessle, (6) “*Low back pain prevalence and associated factors in Iranian population: findings from the national health survey*” by A. Biglarian and colleagues, (7) “*Validation of the self-assessment of treatment questionnaire among patients with postherpetic neuralgia*” by K. Wyrwich and colleagues, (8) “*Sarcoidosis and pain caused by small fiber neuropathy*” by L. Heij and colleagues, (9) “*Depressive symptoms, pain, and quality of life among patients with non-alcohol related chronic pancreatitis*” by W. Balliet and colleagues, and (10) “*Depression and anxiety symptoms relate to distinct components of pain experience among patients with breast cancer*” by S. Galloway and colleagues.

As a whole, this body of research highlights the impact, incidence, and characteristics of pain in chronic medial illness, as well as opportunities to improve care and assessment.

*Justin Brown* *Justin Brown* *Jarred Younger* *Jarred Younger* *Alok Madan* *Alok Madan* *Jeffrey Borckardt* *Jeffrey Borckardt*

